# Importance of an Evaluation Phase When Increasing the Occlusal Vertical Dimension: A Systematic Review

**DOI:** 10.1111/jerd.13331

**Published:** 2024-10-15

**Authors:** Jennifer Chantler, Malin Strasding, Franz Strauss, Alexis Ioannidis, Nadja Naenni

**Affiliations:** ^1^ Clinic of Reconstructive Dentistry, Center for Dental Medicine University of Zurich Zurich Switzerland; ^2^ Division for Fixed Prosthodontics and Biomaterials University Clinic of Dental Medicine, University of Geneva Switzerland; ^3^ Faculty of Health Sciences Universidad Autonoma de Chile Santiago Chile

**Keywords:** evaluation phase, occlusal vertical dimension, patient morbidity, success, worn dentition

## Abstract

**Objective:**

To assess whether an evaluation period is necessary for patient and clinical success when increasing the occlusal vertical dimension (OVD) for a full mouth rehabilitation.

**Materials and Methods:**

A systematic search was conducted in six databases: MEDLINE, Web of Science, Scopus, CENTRAL, VHL, and EMBASE. The eligibility criteria of this systematic review used the PICO framework to address the following research question: “In dentate adults requiring an increase in occlusal vertical dimension (OVD) (P), is an evaluation period of the new OVD (I) superior to no evaluation period (C) in terms of success (O)?” Study characteristics, survival, and success rates were extracted from each article. No language restrictions were applied. Study quality was appraised using Cochrane's Risk of Bias 2 tool and Newcastle–Ottawa Scale (NOS) according to the study design.

**Results:**

The electronic search yielded 1188 titles after duplicates were removed. One RCT and 103 non‐comparative articles were found relevant to the search question. Out of the 103 articles, 80 had an evaluation phase and 23 did not. The included RCT revealed that removable devices tended to cause chewing difficulties, unclear speech, and esthetic discomfort. Therefore, the use of a removable appliance to functionally or esthetically evaluate OVD was not indicated prior to the definitive treatment. Esthetics was the highest reported parameter preoperatively for the non‐comparative studies, at 85% with an evaluation and 86% without.

**Conclusion:**

At present, there is lack of evidence that an evaluation period improves clinical and patient‐reported outcomes when increasing OVD for full mouth rehabilitations. Thus, an increase in OVD can be successful with or without an evaluation phase.

**Clinical Significance:**

The evaluation phase helps the clinician manage patient expectations and assist with the treatment sequencing. This phase is most effective with fixed restoration, such as temporary crowns or adhesive restorations. However, there is limited evidence that this phase improves clinical or patient‐reported outcomes.

## 1 Introduction

Loss of tooth structure is a common problem in modern society [1]. With preventative measures such as fluoride and patient education being important reasons for the avoidance to tooth loss, many patients today are able to keep their teeth up into old age [1]. The increased retention of teeth increases the propensity for tooth wear [2]. Patients with loss of tooth structure due to wear often present with discomfort, sensitivity issues, lack of functional ability, or poor esthetics. This group of patients needs to be restored by means of comprehensive restorative rehabilitation and may often require altering of the occlusal vertical dimension (OVD) [3].

Clinicians chose to increase the OVD to attain clinical advantages, such as the enhancement of tooth display, anterior guidance, minimalization of occlusal tooth preparation, and the avoidance of endodontic treatment [4, 5]. Assessment of the loss of OVD in a functional and esthetic manner is critical before any comprehensive rehabilitation since if it is not done correctly, clinical errors within the above parameters may result in unplanned or even more detrimental consequences [6]. A thorough assessment includes both objective parameters—like the need to provide the appropriate thickness for the restorative material and establish favorable incisal and occlusal relationships—as well as subjective parameters such as pink and white esthetics, facial harmony, speech articulation, and comfort [7].

Clinical experience shows that the stomatognathic system has the ability to adapt even to large changes in occlusion and vertical dimension [8, 9]. The mechanism of action, however, is not well understood and clinicians rely on anecdotal evidence to guide their clinical treatment. Currently, there are no clear clinical guidelines that would advise clinicians in determining the correct extent of increase in OVD when treating patients. Intermediate restorations or transitional appliances have been suggested to precede final restorations to attain successful outcomes [7]. These interim provisionals enable clinicians to check whether patients are tolerant to the proposed new OVD [10]. With this technique, potential functional deficits could be rectified while achieving good esthetic results.

Whether a new OVD needs to be evaluated for a certain period of time remains unanswered since the practice is not based upon clear evidence‐based results [3]. Therefore, the current systematic review aims to assess whether an evaluation period is necessary for patient and clinical success when increasing the OVD for a full mouth rehabilitation.

## 2 Materials And Methods

This systematic review reports according to the Preferred Reporting Items for Systematic reviews and Meta‐Analyses 2020 guidelines [11]. A detailed protocol was designed and registered on PROSPERO database (CRD42024473046) before the start of this study.

The topic of increasing OVD is associated with a wide heterogeneity in terms of terminology. Hence, authors have employed the use of the following definitions throughout this article.

### 2.1 Occlusal Vertical Dimension

The glossary of prosthodontic terms defines OVD as the distance measured between two points when the mandibular teeth and the maxillary teeth are in contact [12].

### 2.2 Evaluation Period

The amount of time the clinician deems long enough for the patient to “adapt” to the new OVD by means of interim restorations or a prosthesis (fixed or removable). The terms “testing phase,” “adaptation,” or “stabilization period” have also been used. Regarding inclusion for this systematic review, an article was considered to have an evaluation period if one of the above terms was mentioned as part of its methodology.

### 2.3 Time Points: Preoperative, Interoperative, and Postoperative

Preoperative refers to the initial assessment, before or during the diagnostic phase. Interoperative is at any time point once treatment has started and a postoperative assessment is any review appointment at any time once the procedure is considered completed.

### 2.4 Eligibility Criteria

According to the PICO framework, a focused question was utilized to facilitate the inclusion and exclusion of the studies: “In dentate adults requiring an increase in occlusal vertical dimension (OVD) (P), is an evaluation period of the new OVD (I) superior to no evaluation period (C) in terms of success (O)?”

(P) Participants: human subjects, dentate, requiring an increase of their OVD, and full mouth rehabilitation.

(I) Interventions: evaluation period of new OVD.

(C) Comparison: no evaluation period of new OVD.

(O) Outcome measures: success of the procedure as defined above, including aspects of patient morbidity, comfort, absence of pain, function, no impairment of speech, asymptomatic temporomandibular joint (TMJ) function, and satisfaction with esthetics.

(S) Studies. Randomized controlled clinical trials (RCTs), prospective and retrospective, case–control studies, case reports. No exclusion based on language, date of publication, publication status, length of follow‐up, or number of included patients or arches.

Inclusion Criteria

‐ Human dentate adults after completion of tooth eruption (excluding wisdom teeth and supernumeraries).

Exclusion Criteria

‐ Edentulous arches.
‐ Complete implant supported prosthesis, fixed, or removable.
‐ Patients restored utilizing tooth movement or orthodontic treatment, including Dahl concepts.
‐ Removable final prostheses.

### 2.5 Methods for Identification of Studies

The electronic literature search was carried out in duplicate by two independent authors (JC and MS) analyzing five databases: MEDLINE (via PubMed), Web of Science, Scopus, CENTRAL, and EMBASE from outset to February 9, 2024. No restrictions were applied in terms of language, status or publication date. The complete search strategies are detailed in Data S1.

### 2.6 Study Selection

The titles and abstracts were screened independently by two calibrated review authors (JC and MS) on Rayyan (https://rayyan.ai) [13]. The full‐text of any article meeting the inclusion criteria or with insufficient information in its title and abstract to make a clear decision was then analyzed by the same reviewers. Any disagreements were resolved by discussion with a third reviewer (NN). All studies meeting the inclusion criteria were included and underwent data extraction and assessment of risk of bias.

### 2.7 Data Extraction and Management

Two reviewers (JC and MS) extracted the information from all included studies into a data extraction table (Excel, Microsoft). Disagreements were resolved by discussion and the reasons for exclusion were recorded.

For each study, the following data were recorded:

‐ General information: title, author, year, journal, study design, aim of study.
‐ Methods and population: sample size, inclusion and exclusion criteria, gender (male, female), age (mean).
‐ Exposure: amount of OVD increased and duration of the evaluation period.
‐ Outcomes: comments on patients' comfort, speech, function, esthetics, TMJ, and pain at three time points: preoperative, intraoperative, and postoperative.
‐ Risk of bias assessment

The risk of bias was assessed in duplicate as part of the data extraction process, using the recommended Cochrane Risk of Bias 2 tool for RCT 0.14. Only few articles specifically looked at the stated PICO question. For the selected prospective and retrospective articles, an article was considered to have a similar risk of bias to a case series if it did not report specifically according to the PICO question.

### 2.8 Data Analysis

The singularly included RCT did not permit a meta‐analysis to assess the importance of an evaluation phase when increasing OVD. A narrative synthesis was employed for this review, as a meta‐analysis was deemed inappropriate due to the significant heterogeneity among the remaining studies. The synthesis process started with a preliminary analysis, during which data were extracted and the outcomes organized into tabular form. This method provided a comprehensive summary of the findings and facilitated the identification of potential patterns within the data.

Success was defined as the patient's ability to accept the received treatment. Published authors on this topic commonly refer to a successful change in OVD as the patient was “comfortable,” “functional,” not in any pain from sensitivity or from the masticatory system, and TMJ. These are the aspects which many consider important for a successful outcome [6]. Articles had to report on patients' morbidities at the following time points: preoperative; intraoperatively, after an evaluation phase; postoperative; to be included in the search.

## 3 Results

### 3.1 Study Selection

**FIGURE 1:**
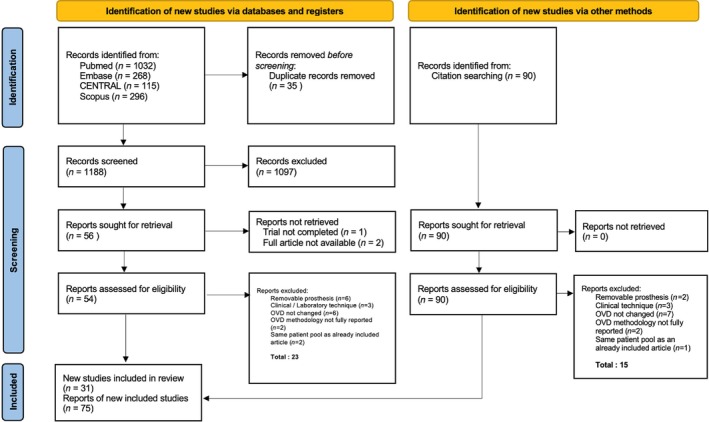
PRISMA 2020 flow diagram of included searches of databases, registers, and other sources [11].

The initial electronic database search yielded a total of 1878 entries, of which 1032 were retrieved from Medline (via PubMed), 115 from CENTRAL, 167 from Scopus, 296 from Web of Science, and 268 from Embase. After excluding 725 duplicates, the total number of entries was 1153. Of these, 1097 articles were discarded after reviewing the titles and abstracts. An additional 73 articles were identified through cross‐reference checking. In total, 129 publications were selected for full‐text analysis and 26 were excluded during this period. A flowchart that depicts the selection process is displayed in Figure 1.

Finally, 104 publications, including one RCT reporting on a combined number of 536 participants met the inclusion criteria and were incorporated in this systematic review.

### 3.2 Characteristics of the Included Studies

Table 1 highlights the extracted results of the included RCT [10]. The authors were able to perform a RCT evaluating the need to test an increase in OVD prior to restorative treatment. Patients were randomly divided into two groups and in one group, individuals were wearing a clear acrylic removable appliance during an evaluation phase, whereas in the second group, no evaluation phase was applied. Removable appliances were worn 24 h a day for a 3‐week period, which can be considered as a reversible way to test the new OVD. The final restorative material used in both groups and for all teeth was composite resin. Patients with the removable device found difficulties in chewing, unclear speech, and discomfort about their appearance. The authors concluded that the symptoms did not reflect the patient's ability to adapt to the OVD increase but was likely related to the poor characteristics of the appliance, poor stability, and contours. With these symptoms intraoperatively, this presented difficulties for the clinicians when attempting to perform the treatment. As such, the use of a removable appliance to functionally or esthetically evaluate OVD was not indicated prior to the definitive treatment.

**TABLE 1:** Extracted data of RCT.

Author	Number of patients	Test group	Control	Test group evaluation period	Definitive restorative material	Outcome measured	Preoperative assessment						Interoperative assessment (for evaluation group)						Postoperative assessment					
							C	S	F	E	T	P	C	S	F	E	T	P	C	S	F	E	T	P
Crins, Opdam et al. 2023 [10]	42	Clear acrylic occlusal splint	No occlusal splint	3 weeks	Composite resin	OHIP‐46	C	S	F	E	T	P	C	S	F	E	T	P	C	S	F	E	T	P

*Note:* Preoperative, interoperative, and postoperative assessments. Where the box is shaded blue, the assessment was performed by clinicians. The orange shading indicates the assessed outcome by clinicians were symptomatic.

Abbreviations: C: comfort; E: esthetics; F: function; P: pain; S: speech; T: temporomandibular joint dysfunction.

Of the non‐comparative articles, 23 did not have an evaluation phase (Table 2). However, all but two articles performed some sort of esthetic evaluation utilizing a direct “mock‐up.” Two articles performed an esthetic try‐in relying solely on a digital face scan [22, 28]. The most common restorative materials utilized in the included studies were direct composite resin, lithium disilicate, and computer aided design and manufactured (CAD/CAM) composite resin. None of the articles reported adverse patient response postoperatively in terms of increased levels of pain, discomfort, dysfunction, poor esthetics, depleted levels of speech, or issues with the TMJ.

**TABLE 2:** Prospective/case reports/case series (no evaluation phase).

Author	Number of patients	Amount of OVD opened	No evaluation phase definitive material	Follow‐up time of postoperative assessment (months)	Preoperative assessment						Interoperative assessment						Postoperative assessment					
					C	S	F	E	T	P	C	S	F	E	T	P	C	S	F	E	T	P
Del Curto, Saratti et al. 2018 [14]	1	1.2 mm at the incisal pin	Indirect CAD/CAM composite resin CeraSmart, GC (posterior teeth) Direct Microhybrid composite resin Tetric Evoceram (anterior teeth)	3			F	E	T											E		
Guess Gierthmuehlen and Steger 2016 [15]	1	NR	Indirect Lithium disilicate IPS e.max Press, Ivoclar Vivadent	18			F	E	T	P										E	T	P
Mehta, Lima et al. 2021 [16]	34	3 mm posteriorly	Direct Microhybrid composite resin Clearfil AP‐X, Kuraray Nanohybrid composite resin IPS Empress Direct, Invoclar Vivadent	1, 12, 36, 60	C		F	E	T	P							C	S	F	E		P
Oudkerk, Eldafrawy et al. 2020 [17]	7	5.5 mm at incisal pin	Indirect PICN Vita Enamic	1, 6, 12, 24			F	E	T	P										E		
Cascales, Sauro et al. 2023 [18]	8	NR	Indirect CAD/CAM composite resin Cerasmart GC Lithium disilicate IPS e.max Press, Ivoclar Vivadent Direct Composite resin	60			F	E											F	E		
Maier, Crins et al. 2024 [19]	22	3 mm posteriorly	Indirect CAD/CAM composite resin Lava Ultimate, 3 M Direct Nanohybrid composite resin Filtek Supreme XTE, 3 M	1, 12, 36, 60			F	E											F	E		
Hansen 2024 [20]	1	NR	Indirect Lithium disilicate IPS e.max, Ivoclar Vivadent	NR			F	E											F	E		
Ning, Bronkhorst et al. 2022 [21]	38	1.6 mm posteriorly	Direct Nanohybrid composite resin IPS Empress Direct Ivoclar Vivadent Microhybrid composite resin Clearfil AP‐X, Kurrary	1, 60					T	P									F	E		
Blasi Beriain, Rocca et al. 2022 [22]	1	NR	Direct Microhybrid composite resin Inspiro Skin White Edelweiss	NR			F		T	P									F	E		
Crins, Opdam et al. 2022 [23]	22	3.5 mm anteriorly	Indirect CAD/CAM composite resin Lava Ultimate, 3 M	1			F	E		P									F	E	T	
Fradeani, Bacherini et al. 2021 [24]	45	3 mm anteriorly	Indirect Lithium disilicate IPS e.max, Ivoclar Vivadent	3, 6, 12				E									C		F	E		
Villavicencio‐Espinoza, Giacomini et al. 2020 [25]	1	NR	Direct Microhybrid Esthet X, Dentsply	1, 6, 36, 48	C		F	E		P							C		F	E	T	
Sterenborg, Kalaykova et al. 2020 [26]	24	3 mm posteriorly	Indirect CAD/CAM composite resin Lava Ultimate, 3 M	1		S		E										S				
Moreira, Freitas et al. 2019 [27]	1	NR	Indirect Zirconia oxide Prettau Zirkon, Zirkonzahn Lithium disilicate IPS e.max press/CAD, Ivoclar Vivadent	48				E									C		F	E		
Ferrando‐Cascales, Astudillo‐Rubio et al. 2020 [28]	1	NR	Indirect CAD/CAM hybrid ceramic Grandio Blocs HT, VOCO	NR	C			E		P									F	E		
Negrão, Cardoso et al. 2018 [29]	1	NR	Direct Microhybrid	NR				E	T	P							C			E		
Stewart 2017 [30]	1	NR	Direct Nanocomposite Gaeial Universial Flo	NR	C		F	E		P							C		F	E	T	
Bartlett and Varma 2017 [31]	35	NR	Direct Microhybrid composite resin Duo Ceram, Dentsply	1				E											F			
Duffield 2016 [32]	1	NR	Indirect Lithium disilicate IPS e.max Press	1			F	E														
Metz, Stapleton et al. 2015 [33]	1	4.5 mm anteriorly	Indirect Compsoite resin Radica, Dentsply	3			F	E											F	E	T	
Ramseyer, Helbling et al. 2015 [34]	7	3 mm from the incisal pin	Direct Compsoite resin CeramX, Dentsply	NR	C					P							C					P
Derchi, Vano et al. 2015 [35]	1	NR	Direct Compsoite resin Estenia C and B, Kuraray	1, 6			F	E	T										F			
Broliato, Volcato et al. 2008 [36]	1	2 mm fron the incisal pin	Direct Microhybrid compsoite resin Filtek Z250, 3 M	0.5				E											F	E		

*Note:* Preoperative, interoperative, and postoperative assessments. Where the box is shaded blue, the assessment was performed by clinicians. The orange shading indicates the assessed outcome by clinicians were symptomatic.

Abbreviations: C: comfort; E: esthetics; F: function; NR: not reported; P: pain; S: speech; T: temporomandibular joint dysfunction.

Out of the 103 non‐comparative studies, 80 articles investigated on an evaluation phase (Table 3). Of these, 19 articles utilized a removable device. The majority of articles utilized a clear acrylic splint and two groups utilized a tooth‐colored and shaped CAD/CAM polycarbonate splint [54, 115]. Forty‐nine articles reported on fixed solutions using direct composite resin or temporary crown restorations made from PMMA or bisacrylic resin. A mixed modality was reported in nine articles, where patients started off with a removable device for a period of time before transferring to a fixed temporary solution. Three articles reported on a combination of both removable and fixed devices at the same time to assess the OVD. Out of the 80 articles, only two reported on esthetic concerns within the evaluation phase (interoperative). These issues were resolved posttreatment. Twenty‐five articles transitioned to segmented final restorations, 35 transitioned full arch, and 20 did not report specifically.

**TABLE 3:** Prospective/case reports/case series (evaluation phase).

Author	Number of patients	Amount of OVD opened	Evaluation phase material/appliance	Follow‐up time of evaluation phase (months)	Preoperative assessment						Interoperative assessment						Postoperative assessment					
					C	S	F	E	T	P	C	S	F	E	T	P	C	S	F	E	T	P
Canger, Celenk et al. 2010 [37]	1	3 mm posteriorly	Removable Occlusal splint	4				E			C				T	P			F	E		
Edelhoff, Güth et al. 2019 [38]	7	NR	Fixed CAD/CAM PMMA Removable Occlusal splint	3			F	E		P				E			C		F	E		P
LeSage 2020 [39]	1	2.5 mm from the incisal pin	Fixed CAD/CAM composite resin Laval Ultimate, Paradigm MZ100, 3 M Telio and Tetric CAD	3–12			F	E			C	S	F	E			C		F	E		
Edelhoff, Beuwer et al. 2012 [40]	1	NR	Removable Occlusal splint	12				E		P			F	E					F	E		
Nazeer, Ghafoor et al. 2020 [41]	1	NR	Fixed Direct composite resin + indirect acrylic bridges	3	C		F	E		P	C			E	T	P	C			E		P
Zeighami, Siadat et al. 2015 [42]	1	1 mm anteriorly	Fixed Indirect acrylic temporaries	2		S	F		T	P		S							F	E		P
Kim, Lee et al. 2016 [43]	1	2 mm anteriorly	Fixed Indirect acrylic temporaries	1					T	P		S		E	T			S	F			P
Jain and Sindhu 2017 [44]	1	4 mm posteriorly	Removable Occlusal splint Fixed Indirect acrylic temporaries	3	C	S	F	E	T	P		S		E	T		C		F	E		
Moreira, Freitas et al. 2018 [45]	1	4 mm anteriorly	Fixed Indirect metal acrylic temporaries	3			F	E	T	P				E	T					E		
Jain and Janani 2016 [46]	1	3 mm anteriorly	Removable Acrylic denture Fixed Indirect acrylic temporaries	3			F	E		P		S	F	E					F	E		
Jain, Nallaswamy et al. 2013 [47]	1	4 mm from the incisal pin	Fixed Indirect acrylic temporaries	3		S	F	E	T	P	C	S	F	E	T	P			F	E		
Kang, Heo et al. 2018 [48]	1	1.5 mm anteriorly	Removable Acrylic occlusal splint Fixed Indirect metal acrylic temporaries	3			F	E			C		F	E	T		C			E	T	P
Lee, Joo et al. 2016 [49]	1	3 mm anteriorly	Fixed Direct bisacryl temporaries	3			F	E						E	T	P			F	E		
Yang, Kim et al. 2022 [50]	1	2.5 mm anteriorly	Removable Acrylic occlusal splint	1.5	C	S		E	T			S	F			P			F	E		P
			Fixed Indirect acrylic temporaries	3																		
Kim, Yeo et al. 2022 [51]	1	3 mm anteriorly	Fixed Indirect acrylic temporaries	1.5	C				T				F	E					F	E		
Cha, Yeom et al. 2017 [52]	1	3 mm anteriorly	Fixed Indirect acrylic temporaries	3			F	E	T		C		F	E	T		C			E	T	P
Kim, Jeong et al. 2021 [53]	1	NR	Fixed CAD/CAM indirect acrylic temporaries	4	C		F	E	T	P	C	S	F	E					F	E	T	
Saratti, Merheb et al. 2020 [54]	1	1.25 mm anteriorly	Removable CAD/CAM printed indirect mock‐up	1			F	E	T	P		S	F	E						E	T	P
Ergun and Ataol 2018 [55]	1	5 mm anteriorly	Fixed Indirect acrylic temporaries	3				A		P		S			T	P	C	S	F	E		P
Wilkins 2016 [56]	1	NR	Fixed Indirect resin composite onlays	4				E						E		P	C		F	E		
Liebermann, Frei et al. 2018 [57]	1	5 mm anteriorly	Fixed Indirect acrylic temporaries	4			F	E					F							E		
Liebermann, Rafael et al. 2017 [58]	1	3.7 mm from the incisal pin	Fixed Direct bisacryl temporaries	NR			F	E				S		E					F			
Dadarwal, Sharma et al. 2023 [59]	1	4 mm from the incisal pin	Removable Acrylic occlusal splint	1.5	C	S	F	E	T	P			F						F	E		
Stumbaum, Konec et al. 2010 [60]	1	5 mm anteriorly	Removable Acrylic occlusal splint	2	C				T	P						P	C					P
Saeidi Pour, Edelhoff et al. 2015 [61]	1	2.1 mm anteriorly	Removable Acrylic occlusal splint	3			F	E	T	P	C					P			F	E		
El‐Kerdani and Nimmo 2016 [62]	1	4 mm from the incisal pin	Removable Acrylic occlusal splint	0.75				E					F		T				F			
Malkoc, Sevimay et al. 2009 [63]	1	NR	Removable Acrylic occlusal splint Fixed Direct bisacryl temporaries	1	C		F	E		P					T		C				T	
Badalotti, Lenz et al. 2023 [64]	1	2 mm posteriorly	Fixed Direct bisacryl temporaries	1				E	T			S	F	E	T	P			F	E		
Kumar, Reddy et al. 2023 [65]	1	1.5 mm from the incisal pin	Fixed Direct bisacryl temporaries	0.75					T	P	C					P	C		F			
Albertini, Bechelli et al. 2023 [66]	1	NR	Fixed Direct composite temporaries	4			F	E				S		E						E		
Luna‐Domínguez, Luna‐Domínguez et al. 2023 [67]	1	3 mm anteriorly	Fixed Direct bisacryl temporaries	NR				E					F	E						E		
D'Arcangelo, Vadini et al. 2022 [68]	1	5 mm anteriorly	Removable Acrylic occlusal splint	2				E	T	P				E					F	E		
Farao and Roomaney 2022 [69]	1	2 mm anteriorly	Fixed Direct composite resin	6			F	E	T			S		E					F	E		
Lippert, Andrade et al. 2022 [70]	1	3 mm anteriorly	Removable Acrylic occlusal splint	1			F	E				S		E					F	E		
Edelhoff, Erdelt et al. 2023 [71]	21	NR	Removable Acrylic occlusal splint OR Fixed CAD/CAM indirect PMMA temporaries	3			F	E				S		E						E		
Durrani, Pandey et al. 2022 [72]	1	3 mm anteriorly	Fixed Indirect acrylic temporaries	4	C		F	E	T			S	F	E					F	E		
Hasanzade, Ghodsi et al. 2021 [73]	1	2 mm anteriorly	Fixed Direct composite resin	5	C		F	E		P	C		F				C		F	E		
Tauböck, Schmidlin et al. 2021 [74]	13	NR	Removable Acrylic occlusal splint	6			F	E							T		C		F			
Torosyan, Vailati et al. 2022 [75]	45	NR	Fixed Direct composite resin	1				E			C	S	F	E	T		C		F	E	T	P
Dallari, Monaco et al. 2019 [76]	1	2	Removable Acrylic occlusal splint	0.5	C	S		E	T	P				E	T					E	T	P
Cardenas‐Sallhue, Delgadillo‐Avila et al. 2020 [77]	1	4	Fixed Direct bisacryl temporaries	0.75	C			E		P	C		F	E	T		C		F	E	T	
Maharjan, Joshi et al. 2019 [78]	1	4	Removable Acrylic occlusal splint	1	C		F	E		P	C		F	E	T							
Taha, Abu‐Elfadl et al. 2021 [79]	1	6	Fixed Direct bisacryl temporaries	3	C		F	E						E			C		F	E	T	P
Lee, Kim et al. 2019 [80]	1	2	Fixed Indirect acrylic temporaries	8				E			C		F	E	T					E		
Viana, do Amaral et al. 2020 [81]	1	5	Fixed Direct bisacryl temporaries	2					T	P	C			E	T	P	C		F			
Yang, Liu et al. 2019 [82]	1	NR	Fixed Direct bisacryl temporaries	2		S	F	E		P		S	F	E	T		C			E		P
Mak and Chio 2019 [83]	1	NR	Fixed Direct bisacryl temporaries	1	C		F	E		P			F	E					F	E		
Ammannato, Rondoni et al. 2018 [84]	1	NR	Fixed Direct bisacryl temporaries	Few weeks				E	T	P		S		E	T			S	F	E		
Pour, Engler et al. 2018 [85]	1	2.5	Fixed Direct autopolymerised resin temporaries	3	C		F	E	T	P		S	F			P						
Liebermann, Edelhoff et al. 2019 [86]	29	4	Fixed Indirect CAD/CAM polycarbonate	NR	C	S	F	E		P							C	S	F	E		P
Vahidi 2019 [87]	1	7	Fixed Direct bisacryl temporaries	3			F					S	F	E			C		F	E		
Sterenborg, Maal et al. 2018 [88]	44	1.5	Fixed Direct bisacryl temporaries	NR	C			E									C			E		
Resende, Reis et al. 2018 [89]	1	1.6	Fixed Direct poly methyl methacrylate resin temporaries	0.5				E		P	C											
Sarattia, Del Curtob et al. 2017 [90]	1	1.5	Fixed Direct composite resin	NR	C		F	E	T			S	F	E					F	E		
Klink, Groten et al. 2018 [91]	17	4	Removable Acrylic occlusal splint	4			F	E									C	S	F	E		P
Fisselier and Comut 2018 [92]	1	3	Fixed Direct bisacryl resin and indirect PMMA temporaries	NR	C			E	T	P		S		E			C		F			
Abou‐Ayash, Boldt et al. 2017 [93]	1	3	Fixed Indirect CAD/CAM PMMA temporaries	3				E					F	E	T				F			
Zhao, Gao et al. 2017 [94]	1	10	Removable Acrylic occlusal splint	3			F		T	P					T	P	C		F		T	
Ting, Shuhui et al. 2017 [95]	2	3	Removable Acrylic occlusal splint	3			F		T	P					T	P						
Schlichting, Resende et al. 2016 [96]	1	2	Fixed Direct PMMA temporaries	NR						P				E			C		F	E		
Klink and Huettig 2016 [97]	1	NR	Removable Acrylic occlusal splint	6	C		F	E	T	P		S		E			C		F	E		
Giannuzzi and Motlagh 2015 [98]	1	3	Fixed Direct bisacryl resin	3		S		E			C		F	E					F	E		
Kois and Kois 2015 [99]	1	4.5	Removable Acrylic occlusal splint	0.25	C		F	E	T	P			F	E	T		C		F	E		
Gargari, Lorè et al. 2014 [100]	1	3	Fixed Indirect PMMA temporaries	1	C		F	E		P	C	S	F	E			C	S	F	E		P
Nam and Tokutomi 2015 [101]	1	4	Fixed Indirect PMMA temporaries	3	C		F			P	C	S	F									
Bahillo, Jané et al. 2014 [102]	1	6	Fixed Direct composite resin	1			F	E	T	P	C		F		T	P	C		F	E		
Prasad, Kuracina et al. 2008 [103]	1	3	Fixed Indirect base metal onlays and composite resin	2			F	E			C	S			T				F	E		
Machado, Fonseca et al. 2007 [104]	1	NR	Removable Acrylic occlusal splint	3				E	T						T					E	T	P
Grütter and Vailati 2013) [105]	1	NR	Fixed Direct bisacryl resin	1			F	E	T	P	C	S	F	E			C			E		
Chekhani, Mikeli et al. 2013 [106]	1	3	Removable Acrylic occlusal splint	1			F	E	T	P			F		T	P	C			E	T	P
			Fixed Direct bisacryl resin	4																		
Vailati, Gruetter et al. 2013 [107]	12	NR	Fixed Direct composite resin	1	C		F	E	T	P							C		F	E	T	P
Güth, Silva et al. 2011 [108]	1	7	Removable Acrylic occlusal splint	9				E			C		F						F		T	P
Fradeani, Bacherini et al. 2021 [24]	1	3	Fixed Direct composite resin	1			F	E	T	P			F	E					F	E		
Schwarz, Kreuter et al. 2011 [109]	1	NR	Removable Acrylic occlusal splint Fixed Direct composite resin	3	C		F	E	T	P			F			P	C			E		
Bynum 2010 [110]	1	NR	Removable Acrylic occlusal splint Fixed Direct composite resin	9	C			E	T	P	C		F	E	T		C		F	E	T	
Kumar, Patil et al. 2010 [111]	1	5	Removable Acrylic occlusal splint Fixed Indirect PMMA temporaries	1.5				E	T	P			F	E			C			E		T
Cengiz, Cengiz et al. 2009 [112]	1	3	Removable Acrylic occlusal splint	1			F	E							T	P	C					
Garcia, Sundfeld et al. 2009 [113]	1	NR	Removable Acrylic occlusal splint	NR	C				T	P			F		T			S			T	P
Mizrahi 2008 [114]	1	NR	Fixed Indirect PMMA temporaries	2				E						E		P				E		
Edelhoff, Schweiger et al. 2017 [115]	1	4	Removable CAD/CAM polycarbonate “tooth” splints	NR		S	F	E	T	P	C		F	E								

*Note:* Preoperative, interoperative, and postoperative assessments. Where the box is shaded blue, the assessment was performed by clinicians. The orange shading indicates the assessed outcome by clinicians were symptomatic.

Abbreviations: C: comfort; E: esthetics; F: function; NR: not reported; P: pain; S: speech; T: temporomandibular joint dysfunction.

Sixty‐nine of the non‐comparative articles reported an increase in OVD. Nineteen articles recorded the increase in OVD at the incisal pin giving an average of 3.7 ± 1.8 mm, 37 articles measured between the maxillary and mandibular incisors giving a mean increase of 3.2 ± 1.3 mm, and 13 articles measured from the first molars mentioning an average of 2.6 ± 1.2 mm. The average evaluation time frame was 3.0 ± 2.2 months [0.25–12].

### 3.3 Risk of Bias

**FIGURE 2:**
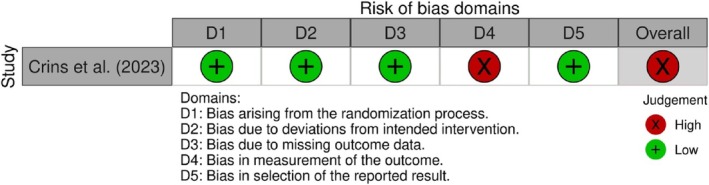
Risk of bias summary of the RCT study [116].

A high risk of bias was recorded for the singular RCT (Figure 2). The reason for this was because of the discrepancy between the two trial groups. The patients had knowledge of the intervention received which had a high chance of influencing the overall outcome [10]. All 103 noncomparable articles were deemed to have the lowest level of evidence due to the inconsistent nature in reporting.

### 3.4 Success Outcomes

Table 4 displays the numbers of how much each outcome was mentioned in each article during the preoperative, intraoperative, and postoperative phase.

**TABLE 4:** Summary of the preoperative, intraoperative, postoperative outcomes, excluding RCT study.

Type of study	Number of articles	Preoperative						Intraoperative						Postoperative					
		C	S	F	E	T	P	C	S	F	E	T	P	C	S	F	E	T	P
Evaluation phase	80	28	10	49	68	39	47	26	28	41	50	32	21	35	6	53	60	15	23
Evaluation phase (with symptoms)		3	2	30	60	6	28	—	—	—	2	—	—	—	—	—	—	—	—
No evaluation phase	23	5	1	14	20	8	11	—	—	—	—	—	—	8	2	15	18	5	3
No evaluation phase (with symptoms)		0	0	4	12	0	4	—	—	—	—	—	—	—	—	—	—	—	—

*Note:* It is easier for the eye to read. Orange shade refers to data/patients ‘with’ symptoms.

#### 3.4.1 Comfort

Comfort was reported in 32% of cases preoperatively and in 42% postoperatively out of all included articles. Only 3% reported discomfort preoperatively and all the reported discomfort was resolved by the postoperative review.

#### 3.4.2 Speech

Speech was the least reported outcome pre‐ and postoperatively. Studies with an evaluation phase assessed speech in 35% of the articles.

One study aimed to evaluate changes to speech characteristics before and after adaptation of the OVD (without an evaluation phase) [26]. Speech was assessed utilizing speech recordings. Because speech characteristics are variable within the same person, it is considered a subjective assessment. Before treatment, many patients pointed out articulation problems to their dentist. The study showed that the /s/, /f/, /v/, /d/, /t/, /m/ speech sounds decreased when the OVD was increased. For the /t/ and /f/ sounds, the degree of lengthening of the incisors significantly influenced the treatment effect. The acoustic characteristics changed after full mouth composite rehabilitation, but a rebound effect was observed after 1 month of adaptation. Patients also perceived a subjective improvement in speech function after an increase in OVD.

In articles designed with an evaluation phase, the improvement on linguistics had predominantly remained neutral with no changes between baseline and the second OHIP questionnaire, when comparing the speech preoperatively to postoperatively [86].

#### 3.4.3 Function

Function as an outcome was reported preoperatively in 50% of the articles and 54% of those articles reported dysfunction at this time point. Postoperative function was reported in 66% with 0% dysfunction. Within the evaluation phase, function was reported in 51% of the time.

A kinesiograph was utilized in one study in an attempt to assess and quantify function [22]. The device recorded and compared the jaw movements and mapped a patient's envelope of motion before and directly after changing the OVD. Without an evaluation phase, direct composite resin was added to the worn dentition. The use of kinesiography showed that jaw mobility was almost unchanged before and after increase in OVD.

When a visual analogue scale was used to assess patient‐centered outcomes, there was a combined high esthetic and functional acceptance of 94.6% [107]. This study did not have an evaluation phase but there were no reports of muscular discomfort or TMJ dysfunction at any moment before treatment or thereafter [107].

#### 3.4.4 Esthetics

Esthetics was considered one of the most reported parameters preoperatively with 85% of articles mentioning an esthetic assessment, when considering an evaluation phase. In studies without an evaluation phase, it reached 86%. Individuals often presented with perceived impaired esthetics before an increase in OVD. Of those articles with an evaluation phase reporting on an esthetic assessment, 88% mentioned that the esthetics were perceived deficient. Two articles reported on the need for esthetic adjustments during the intraoperative phase before the final delivery of the restorations [53, 84].

A removable clear occlusal splint was unable to provide neither the patient nor the clinician with information on esthetics. In these cases, a direct or indirect esthetic try‐in was required. A tooth‐colored and tooth‐shaped CAD/CAM milled acrylic splint was able to allow patients to assess their esthetics with a removable device [90, 115].

#### 3.4.5 Temporomandibular Joint Dysfunction

Muscular discomfort or TMJ dysfunction was usually assessed by clinicians preoperatively and intraoperatively. No studies without an evaluation phase reported on preexisting temporomandibular dysfunction, whereas studies with an evaluation phase reported on preexisting temporomandibular dysfunction in 8%.

#### 3.4.6 Pain

Preoperatively, pain was frequently reported, often presenting as sensitivity during eating or drinking. This was evident in 55% of the articles. However, no instances of sensitivity or pain were reported during the postoperative review.

## 4 Discussion

The purpose of this systematic review aimed to determine the clinical importance of an evaluation phase when increasing the OVD. The present systematic review predominately revealed:

- Patients are generally adaptable to increases in OVD and can be successfully restored with or without an evaluation phase.
- Tooth‐shaped fixed or removable appliances provide valuable clinical information.
- For full mouth rehabilitation without an evaluation phase, minimally invasive or no preparations should be considered.
- Digital technologies enable consistency and accuracy between the proposed and executed occlusal schemes.

When implementing a new OVD, an evaluation phase gives patients the chance to adapt to the new situation, yet also provides the clinician time to develop confidence and verify the planned outcome [10]. A testing phase was used for evaluation in 80 articles, whereas in 23 articles it was not performed. This shows that even though an evaluation phase is mostly part of the treatment of such complex cases, a considerable number of clinicians will omit this lengthy phase. The verification of the proposed outcome allows clinicians to check a patient's comfort and all the functional and esthetic aspects of the new occlusal scheme. During the try‐in period, a full‐mouth mock‐up can be modified intraorally to optimize the functional and esthetic aspects in response to feedback from the patient [5, 107]. When dentists are inexperienced or lack laboratory support, they are often not able to perform all treatment steps simultaneously due to the constrains of the patient's time and financial considerations. The evaluation phase allows the clinicians to segmentalize and stage the treatment, focusing on fewer clinical variables to reduce the risks of errors. This helps avoid situations where expensive restorations might need to be adjusted or removed.

The new OVD can be evaluated either with the aid of fixed temporary restorations or with removable appliances. Most of the studies employed fixed full coverage temporary restorations for evaluating the increase of OVD. Fixed temporary crown restorations have the advantage of high patient acceptance and comfort, while removable appliances may not be accepted by all patients, especially patients with gag reflex [10]. The removable appliances are a less expensive way of testing the new OVD and mostly transparent acrylic occlusal splints were used for this purpose. One drawback of the clear acrylic splints is the missing esthetic information, as neither the shape nor the color resembles teeth. Thus, an esthetic evaluation of the planned restorations is not possible [10].

To evaluate the esthetics, clinicians should adopt an evaluation technique which delivers more information on the final tooth contour and design. This way, an additional appointment for an esthetic mock‐up is avoided. One study group used CAD/CAM polycarbonate tooth splints, allowing for esthetic evaluation due to the tooth‐colored material and natural tooth outline [115]. While this did increase the cost of the overall treatment, it combined the removable and reversible esthetic prototype in a virtually risk‐free manner [115]. Whenever a solely additive procedure for the full‐mouth rehabilitation is not possible, and teeth must be prepared, a removable appliance may not be sufficient and fixed provisional restorations are indicated. The material used for temporary fixed restorations was mainly direct bisacryl resin and direct composite. Indirect temporary restorations were made of acrylic resin or CAD/CAM fabricated PMMA.

Full coverage temporary restorations were often utilized to control pulpal symptoms during the evaluation phase and subsequently full crowns were delivered as the definitive restorations. However, omitting an evaluation phase was often possible when the clinician did not need tooth preparation. Without reducing the enamel to gain prosthetic space, and the use of minimally invasive restorations helped to avoid sensitivity issues and thus, temporary restorations were not required. The increased OVD reconstruction was restored in an additive manner, with the clinicians designing the restorations to the shape of the lost tooth structure. This is why direct composite resin is a common material in the articles with no evaluation phase. However, 14 articles were able to achieve this with indirect restorations. To assess esthetic parameters such as occlusal plane and length of the anterior teeth, indirect and direct mock‐ups were used before increasing the OVD. There was one study that did not require an esthetic mock‐up as they created a digital patient with the use of a face scan [28].

Conventional analogue semi‐adjustable articulators have been a key instrument for many years. However, the lack of integration between occlusal aspects and the patient's face can jeopardize esthetic references by adding possible errors to the desired planning [66]. New software programs which allow facially driven design and communication among the patient and the dental team can ensure that these errors are avoided. A proposed digital technique for not having an evaluation phase is mainly esthetically guided [66]. The digital consistency and repetition allow fluent networking and communication makes the proposed OVD to be accurately articulated digitally and replicated into the final restorations [3].

The threshold to which the clinician can increase the OVD remains unclear, and it is uncertain whether OVD remains constant throughout an individual's life. Historically, methods for determining OVD have been highly subjective [117]. Previous systematic reviews have also found significant heterogeneity in study designs, a finding consistent with this review. Currently, the general consensus is that a safe increase in OVD is up to 5 mm [117]. With the mean increase in incisal pin measurements from this review at 3.7 ± 1.8 mm, the authors support the conclusion that a 5 mm OVD increase is both safe and predictable.

The present systematic review has limitations. This article focuses on the technical aspects of increasing the OVD. It does not account for clinical decisions about individual patient care while considering clinician's knowledge, skills, attitudes, and preferences. Due to the high subjectivity and high reporting bias of the non‐comparative selective articles, patients with a history of severe medical or psychological issues are often not ideal cases for reporting. In such cases, a thorough anamnesis, pretreatment, and trial phase are essential for building a strong patient–dentist relationship. Ensuring good patient compliance is crucial for a successful treatment in complex cases. An extended evaluation phase or a segmented treatment sequence may be necessary to manage the expectations and establish trust between the patient and dentist.

It is for this reason that designing an RCT for this PICO question can be challenging due to persistent issues of variability inconsistent reporting. The subjectivity of outcomes' measurements significantly complicates the comparison of these studies. For example, the quality of baseline pretreatment assessments or posttreatment assessments varied considerably throughout the article. To improve reporting on OVD acceptance of the OVD, standardized patient‐reported outcome measures should be implemented consistently—preoperatively, during intervention, and postoperatively.

## 5 Conclusions

Within the limitations of this systematic review, it can be concluded that:

1. An increase of the OVD can be successful with or without an evaluation phase.
2. The evaluation phase can be performed with a removable appliance or with provisional or interim restorations. However, removable occlusal splint appliances, however, are not effective for assessing esthetics and can negatively impact patient satisfaction.
3. Esthetics were evaluated routinely at all time points within both groups.
4. The digital workflow enables fabrication of indirect restorations at an increased OVD without the need for an evaluation phase.

## Conflicts of Interest

The authors declare no conflicts of interest.

## Supporting information


Data S1.

## Data Availability

The data that support the findings of this study are available on request from the corresponding author. The data are not publicly available due to privacy or ethical restrictions.
