# Supplementary material for: Importance of an Evaluation Phase When Increasing the Occlusal Vertical Dimension: A Systematic Review
Source: J Esthet Restor Dent. 2024 Oct 15;37(3):669–89. doi: 10.1111/jerd.13331 (PMC12076109; doi:10.1111/jerd.13331)
Supplement: Supplementary file 1 — Data S1. [file JERD-37-669-s001.docx]

PUBMED 9 Feb. 24

| #1 | Tooth loss OR jaw, edentulous OR mouth, edentulous OR full mouth | [44,811](https://pubmed.ncbi.nlm.nih.gov/?term=Tooth+loss+OR+jaw%2C+edentulous+OR+mouth%2C+edentulous+OR+full+mouth&sort=fauth&size=200) |
| --- | --- | --- |
| #2 | Increase occlusal vertical dimension* OR increase vertical dimension occlusion OR occlusion OR rehabilitation OR vertical dimension | [1,066,484](https://pubmed.ncbi.nlm.nih.gov/?term=Increase+occlusal+vertical+dimension%2A+OR+increase+vertical+dimension+occlusion+OR+occlusion+OR+rehabilitation+OR+vertical+dimension&sort=fauth&sort_order=asc&size=200&ac=no) |
| #3 | Evaluation period OR adaptation period OR testing phase OR provisional OR interim | [768,113](https://pubmed.ncbi.nlm.nih.gov/?term=Evaluation+period+OR+adaptation+period+OR+testing+phase+OR+provisional+OR+interim&sort=fauth&sort_order=asc&size=200&ac=no) |
| #4 | Efficacy OR complications OR patient morbidity OR technical failure OR biological complication* OR success OR success rate OR survival OR survival rate OR PROMS OR PROM OR patient outcome* OR cost OR efficiency OR aesthetics OR compliance OR duration | [11,167,121](https://pubmed.ncbi.nlm.nih.gov/?term=Efficacy+OR+complications+OR+patient+morbidity+OR+technical+failure+OR+biological+complication%2A+OR+success+OR+success+rate+OR+survival+OR+survival+rate+OR+PROMS+OR+PROM+OR+patient+outcome%2A+OR+cost+OR+efficiency+OR+aesthetics+OR+compliance+OR+duration&sort=fauth&size=200) |
| #5 | #1 AND #2 AND #3 AND #4 | [1,032](https://pubmed.ncbi.nlm.nih.gov/?term=%234+AND+%232+AND+%233+AND+%235&sort=fauth&size=200) |

Embase

| #1 | 'Tooth loss' OR 'jaw, edentulous' OR 'mouth, edentulous' OR 'full mouth' | [141,327](https://www.embase.com/) |
| --- | --- | --- |
| #2 | 'increase occlusal vertical dimension*' OR 'increase vertical dimension occlusion' OR ‘occlusion’ OR ‘rehabilitation’ OR 'vertical dimension' | [1,190,920](https://www.embase.com/) |
| #3 | ‘evaluation period’ OR ‘adaptation period’ OR ‘testing phase’ OR 'provisional' OR 'interim' | [75,804](https://www.embase.com/) |
| #4 | ‘Efficacy’ OR ‘complications’ OR 'patient morbidity' OR 'technical failure' OR 'biological complication*' OR success OR 'success rate' OR survival OR 'survival rate' OR ‘PROMS’ OR ‘PROM’ OR 'patient outcome*' OR ‘cost’ OR ‘efficiency’ OR ‘aesthetics’ OR ‘compliance’ OR ‘duration’ | [8,570,092](https://www.embase.com/) |
| #5 | #1 AND #2 AND #3 AND #4 | [268](https://www.embase.com/) |

Cochrane CENTRAL

| #1 | MeSH descriptor: [Tooth Loss] explode all trees | 202 |
| --- | --- | --- |
| #2 | MeSH descriptor: [Jaw, Edentulous] explode all trees | 737 |
| #3 | MeSH descriptor: [Mouth, Edentulous] explode all trees | 1024 |
| #4 | Tooth loss OR jaw edentulous OR mouth edentulous OR full mouth | 32449 |
| #5 | #1 OR #2 OR #3 OR #4 | 32449 |
| #6 | ("increase occlusal vertical" NEXT dimension*) OR "increase vertical dimension occlusion" OR occlusion OR rehabilitation OR "vertical dimension" | 100088 |
| #7 | "Evaluation period" OR "adaptation period" OR "testing phase" OR provisional OR interim | 20025 |
| #8 | Efficacy OR complications OR "patient morbidity" OR "technical failure" OR ("biological" NEXT complication*) OR success OR "success rate" OR survival OR "survival rate" OR PROMS OR PROM OR ("patient" NEXT outcome*) OR cost OR efficiency OR aesthetics OR compliance OR duration | 907562 |
| #9 | #5 AND #6 AND #7 AND #8 | 115 |

Scopus

| #1 | TITLE-ABS-KEY ( "tooth loss" OR "jaw, edentulous" OR "mouth, edentulous" OR "full mouth" ) | 23940 |
| --- | --- | --- |
| #2 | TITLE-ABS-KEY ( "increase occlusal vertical dimension*" OR "increase vertical dimension occlusion" OR occlusion OR rehabilitation OR "vertical dimension" ) | 805509 |
| #3 | TITLE-ABS-KEY ( "evaluation period" OR "adaptation period" OR "testing phase" OR provisional OR interim ) | 82885 |
| #4 | TITLE-ABS-KEY ( efficacy OR complications OR "patient morbidity" OR "technical failure" OR "biological complication*" OR success OR "success rate" OR survival OR "survival rate" OR proms OR prom OR "patient outcome*" OR cost OR efficiency OR aesthetics OR compliance OR duration ) | 14,866,101 |
| #5 | #1 AND #2 AND #3 AND #4 | 167 |

Web of Science

| #1 | TS=("Tooth loss" OR "jaw, edentulous" OR "mouth, edentulous" OR "full mouth") | [30,592](https://www.webofscience.com/wos/alldb/summary/becc87ee-35e7-4633-bd81-965c30594c18-cb7c90da/relevance/1) |
| --- | --- | --- |
| #2 | TS=("increase occlusal vertical dimension*" OR "increase vertical dimension occlusion" OR occlusion OR rehabilitation OR "vertical dimension") | [1,117,662](https://www.webofscience.com/wos/alldb/summary/55d16375-b909-402f-8a80-ee72afa8691a-cb619913/relevance/1) |
| #3 | TS=("Evaluation period" OR "adaptation period" OR "testing phase" OR provisional OR interim) | [128,110](https://www.webofscience.com/wos/alldb/summary/a7fd406d-cce1-4d91-b946-18515a330b12-cb619eab/relevance/1) |
| #4 | TS=(Efficacy OR complications OR "patient morbidity" OR "technical failure" OR "biological complication*" OR success OR "success rate" OR survival OR "survival rate" OR PROMS OR PROM OR "patient outcome*" OR cost OR efficiency OR aesthetics OR compliance OR duration) | [[29,154,478](https://www.webofscience.com/wos/alldb/summary/3c6b0692-e636-410d-959e-a69cf515c629-cb7ca3bd/relevance/1)](https://www.scopus.com/search/history/results.uri?origin=searchhistory&shid=7) |
| #5 | #1 AND #2 AND #3 AND #4 | [296](https://www.webofscience.com/wos/alldb/summary/88ebfad3-bd6e-474b-bdf3-f338e87dff49-cb7caff7/relevance/1) |

Total 1878

Duplicates removed : 1153
